# Phosphorylable tyrosine residue 162 in the double-stranded RNA-dependent kinase PKR modulates its interaction with SUMO

**DOI:** 10.1038/s41598-017-12777-7

**Published:** 2017-10-25

**Authors:** Carlos F. de la Cruz-Herrera, Maite Baz-Martínez, Ahmed El Motiam, Santiago Vidal, Manuel Collado, Anxo Vidal, Manuel S. Rodríguez, Mariano Esteban, Carmen Rivas

**Affiliations:** 1 Department of Molecular and Cellular Biology, Centro Nacional de Biotecnología-CSIC, Darwin 3, Madrid, 28049 Spain; 2 Department of Molecular Genetics, University of Toronto, 1 Kings College Circle, Toronto, M5S 1A8 Canada; 3 Centro de Investigación en Medicina Molecular (CIMUS), Universidade de Santiago de Compostela, Instituto de Investigaciones Sanitarias (IDIS), Santiago de Compostela, E15706 Spain; 4 Instituto de Investigación Sanitaria de Santiago de Compostela (IDIS), Complexo Hospitalario Universitario de Santiago de Compostela (CHUS), SERGAS, Santiago de Compostela, E15706 Spain; 5 Departamento de Fisioloxía and Centro de Investigación en Medicina Molecular (CIMUS), Universidade de Santiago de Compostela, Instituto de Investigaciones Sanitarias (IDIS), Santiago de Compostela, E15782 Spain; 6 Advanced Technology Institute in Life Sciences (ITAV) CNRS-USR3505, 31106, Université de Toulouse, UPS, Toulouse, France; 7 Institut de Pharmacologie et de Biologie Structurale (IPBS) CNRS-UMR5089, 31077, Université de Toulouse, UPS, Toulouse, France

## Abstract

Activated dsRNA-dependent serine/threonine kinase PKR phosphorylates the alpha subunit of eukaryotic initiation factor 2 (eIF2α), resulting in a shut-off of general translation, induction of apoptosis, and inhibition of virus replication. PKR can be activated by binding to dsRNA or cellular proteins such as PACT/RAX, or by its conjugation to ISG15 or SUMO. Here, we demonstrate that PKR also interacts with SUMO in a non-covalent manner. We identify the phosphorylable tyrosine residue 162 in PKR (Y162) as a modulator of the PKR-SUMO non-covalent interaction as well as of the PKR SUMOylation. Finally, we show that the efficient SUMO-mediated eIF2α phosphorylation and inhibition of protein synthesis induced by PKR in response to dsRNA depend on this residue. In summary, our data identify a new mechanism of regulation of PKR activity and reinforce the relevance of both, tyrosine phosphorylation and SUMO interaction in controlling the activity of PKR.

## Introduction

The dsRNA-dependent serine/threonine kinase PKR has an essential role in innate immunity to viral infection due to its ability to phosphorylate eIF2α and to inhibit general translation. In addition, PKR has been involved in the regulation of the p53, NFκB, p38MAPK or insulin pathways^1^. PKR is induced by type I interferon and is activated upon binding to dsRNA, which causes the homodimerization and autophosphorylation of PKR at several residues located on both, the N-terminal dsRNA binding domain and the C-terminal kinase domain^2–4^. PKR can also be activated by binding to heparin, PKR-activating protein (PACT), or ISG15^5–7^. Recently, we demonstrated that covalent attachment of small ubiquitin-like modifiers (SUMO) to PKR protein also enhanced the activation of the kinase and contributed to its antiviral activity^8^. SUMOylation of a substrate can be promoted by the presence of SUMO interacting motifs (SIMs) that mediate non-covalent interaction with SUMO^9–12^. SIMs consist of a short core sequence of hydrophobic amino acids (V/I/L)X(V/I/L)(V/I/L), which are frequently flanked by a stretch of acidic residues that may play a role in increasing the affinity or in determining the orientation of the interactions^13,14^.

In this report, we show that PKR can interact with SUMO in a non-covalent manner and we identify the phosphorylable tyrosine residue 162 as a modulator of both, non-covalent and covalent PKR-SUMO interaction. Mimicking tyrosine phosphorylation by mutation of this tyrosine residue to aspartic acid abolished PKR-SUMO interaction, regulated PKR SUMOylation and inhibited the activation of PKR by SUMO. In summary, the results shown here identify a new mechanism involved in the regulation of PKR activity, reinforcing the relevance of tyrosine phosphorylation and SUMO interaction in this process.

## Results and Discussion

### PKR-SUMO interaction is modulated by Y162 in PKR

**Figure 1:**
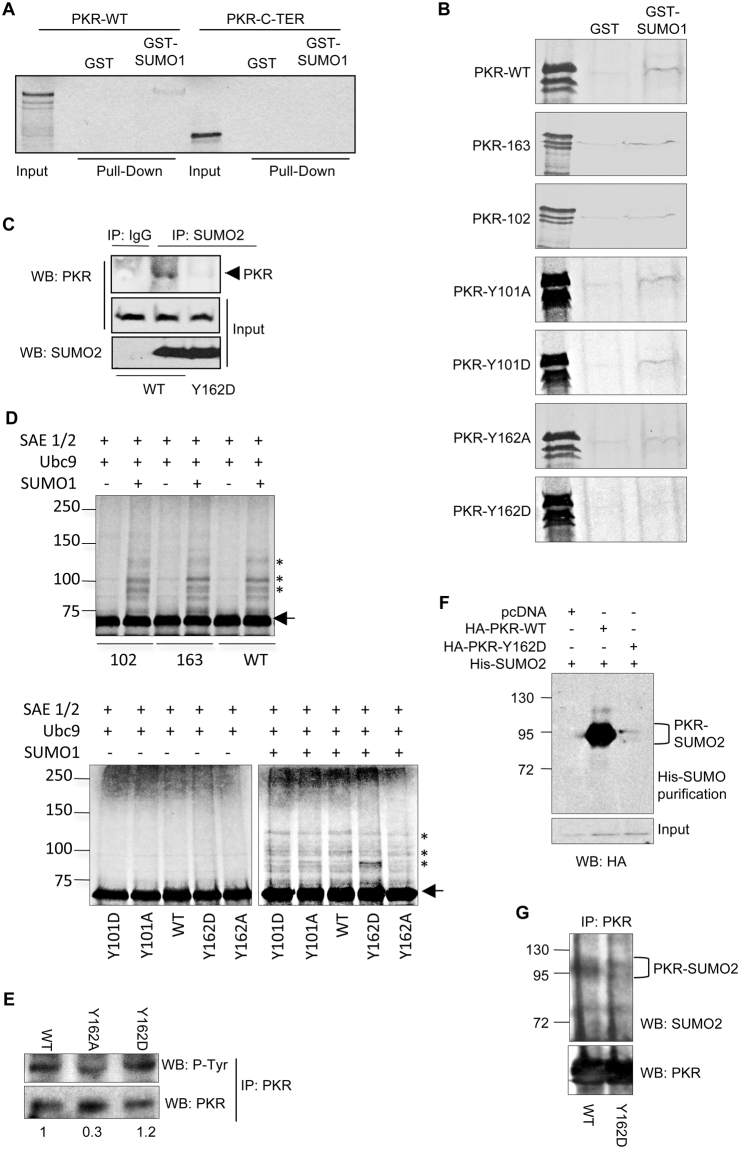
Y162 in PKR modulates the PKR-SUMO covalent and non-covalent interaction. (**A**) Pulldown assay of [^35^S]methionine-labeled *in vitro*-translated PKR protein or the C-terminus of PKR (PKR-C-ter) with GST or GST-SUMO1. (**B**) Pulldown assay of [^35^S]methionine-labeled *in vitro*-translated PKR-WT, PKR mutant in the SIM-102, PKR mutant in the SIM-163, PKR-Y101D, PKR-Y101A, PKR-Y162A or PKR-Y162D with GST or GST-SUMO1. (**C**) PKR−/− cells were co-transfected with PKR-WT or PKR-Y162D and SUMO2. At 36 h after transfection, the protein extracts were immunoprecipitated with anti-SUMO2 antibody. Western-blot analysis of the immunoprecipitated proteins with anti-PKR antibody was then carried out. (**D**) [^35^S]methionine-labeled PKR-WT, the PKR mutants in SIM-102 or SIM163, PKR-Y101A, PKR-Y101D, PKR-Y162A or PKR-Y162D proteins were used as substrates in an *in vitro* SUMOylation assay in the presence of SUMO1. Arrows point to non-SUMOylated PKR protein. Stars indicate the position of PKR-SUMO bands. (**E**) PKR−/− cells were transfected with PKR-WT, PKR-Y162A or PKR-Y162D, and treated with poly(I:C). At 48 h after transfection, the protein extracts were immunoprecipitated with anti-PKR antibody. Western-blot analysis of the immunoprecipitated proteins with anti-phosphotyrosine antibody (P-Tyr) was then carried out. The ratio between tyrosine phosphorylated and total PKR protein is shown below the blots. (**F**) HEK-293 cells were co-transfected with PKR-WT or PKR-Y162D, Ubc9 and His6-SUMO2. At 36 h after transfection, total protein extracts and histidine-purified proteins were analyzed by Western-blot with anti-HA antibody. (**G**) PKR−/− cells were co-transfected with PKR-WT or PKR-Y162D and SUMO2. At 36 h after transfection, the protein extracts were immunoprecipitated with anti-PKR antibody. Western-blot analysis of the immunoprecipitated proteins with anti-SUMO2 antibody was then carried out.

We recently reported that PKR can conjugate to SUMO *in vitro* and in *vivo* ^8^. We also know that some SUMOylated proteins can interact with SUMO in a non-covalent manner^9,12,14–17^. The short core sequence of hydrophobic amino acids (V/I/L)X(V/I/L)(V/I/L) was reported to be the minimal motif needed for SUMO interaction (Song *et al*., 2004). The amino acid sequence of PKR contains 10 domains consisting of hydrophobic residues with this sequence, three located at the N-terminal part of the protein (72VEIL75, 102IGLI105 and 163LQIL166) and 7 at the C-terminal part of PKR (270IELI273, 318VNIV321, 389VLAL392, 420IFLV423, 474LGLI477, 475GLIL478 and 481LLHV484). To evaluate the non-covalent interaction of PKR with SUMO, we performed a GST-pulldown assay using [^35^S]methionine-labeled *in vitro*-translated PKR protein or the C-terminus of PKR (amino acids 266–550) and either GST or GST-SUMO1. As shown in Fig. 1A, PKR was able to interact with GST-SUMO1 but not with GST, indicating that PKR interacts with SUMO1 in a non-covalent manner. We did not observe interaction between GST-SUMO1 and the C-terminus of PKR (Fig. 1A), suggesting that the SUMO-PKR interaction occurs through the N-terminus of PKR.

It has been reported that phosphorylation and/or negative charged amino acids juxtaposed to the hydrophobic core of the SIM can modulate SUMO binding^18–20^. PKR is phosphorylated at several residues and phosphorylation of PKR contributes to regulate its activity. Interestingly, two of the putative SIMs in the N-terminus of PKR, SIM-102 (IGLI) and SIM-163 (LQIL), are preceded by autophosphorylable tyrosine residues (Y101 and Y162, respectively) that have been previously reported to be important for PKR activity^3^. Therefore, we generated PKR mutants in the SIM-102, SIM-163, and in tyrosine residues Y101 and Y162. In the case of tyrosine mutations, these amino acids were changed to alanine or aspartic acid residues, changes that block or mimic tyrosine phosphorylation, respectively. We then evaluated the non-covalent interaction of the mutants with SUMO1 using an *in vitro* GST pulldown assay. We observed that the PKR-SIM102, PKR-SIM163, PKR-Y101D, PKR-Y101A or PKR-Y162A mutants interacted with GST-SUMO1 similarly to the PKR-WT protein (Fig. 1B). However, as shown in Fig. 1B, we did not observe interaction between PKR-Y162D and GST-SUMO1. To confirm these results *in vivo*, PKR-deficient cells (PKR−/−) were co-transfected with SUMO2 and PKR-WT or PKR-Y162D, and 36 h after transfection SUMO2 protein was immunoprecipitated using anti-SUMO2 antibody. As shown in Fig. 1C, anti-SUMO2 antibody immunoprecipitated PKR-WT protein but not the PKR-Y162D mutant protein. These results indicate that the specific substitution of this autophosphorylable tyrosine residue 162 in PKR by aspartic acid inhibits the non-covalent interaction of PKR with SUMO proteins.

For some proteins, the non-covalent interaction with SUMO can mediate their SUMOylation by facilitating the recruitment of SUMO-loaded Ubc9^9,12,14–17^. Since Y162D mutation abolished the non-covalent binding of PKR to SUMO, we decided to analyze whether its SUMOylation is also altered. First, we carried out an *in vitro* SUMOylation assay using [^35^S]methionine-labeled *in vitro*-translated PKR-WT or the PKR mutants PKR-SIM102, PKR-SIM163, PKR-Y101D, PKR-Y101A, PKR-Y162D and PKR-Y162A, as substrates. As reported previously^8^, the addition of SUMO1 to the SUMOylation reaction led to the appearance of at least three PKR-WT-SUMO1 bands of around 85, 105 and 125 kDa (Fig. 1D). A similar pattern of SUMO conjugated products was observed when we employed the PKR mutants in the SIM domains, PKR-Y101D, PKR-Y101A or PKR-Y162A as substrates (Fig. 1D). However, the PKR-Y162D mutant exhibited an altered SUMOylation pattern characterized by a stronger 85 kDa band and reduced intensity of the 105 and 125 kDa bands (Fig. 1D). These results suggested that phosphorylation of the residue 162 modulates the conjugation of SUMO1 to PKR. To verify the phosphorylation of the Y162D mutant, PKR−/− cells were transfected with PKR-WT, PKR-Y162D or PKR-Y162A and the protein immunoprecipitated with anti-PKR antibody was analyzed by Western-blot with anti-phosphotyrosine antibody. As shown in Fig. 1E, the ratio between tyrosine phosphorylated and total PKR protein was higher for the PKR-Y162D mutant than for the PKR-Y162A mutant, and similar to the one detected for PKR-WT protein. To confirm the altered SUMO conjugation of the PKR-Y162D mutant, we co-transfected HEK-293 cells with Ubc9, His6-SUMO2 and pcDNA, HA-PKR-WT or the HA-PKR-Y162D mutant, and 36 h after transfection the histidine-tagged purified proteins were analyzed by Western-blot using anti-HA antibody. As shown in Fig. 1F, we detected the HA-PKR-WT-SUMO2 bands, as expected^8^. In addition, we observed a clear reduction in the SUMO2 modification of the PKR-Y162D mutant (Fig. 1F). Similar results were obtained after analysis of the transfected proteins by immunoprecipitation. For this, PKR-WT or PKR-Y162D transfected in PKR−/− cells were immunoprecipitated with anti-PKR antibody and then analyzed by Western-blot with anti-SUMO2 antibody. We observed bands corresponding to PKR-WT protein modified by SUMO2 that were clearly reduced in the lane corresponding to PKR-Y162D (Fig. 1G), indicating that Y162D mutation inhibited PKR SUMO2 modification and suggesting that the phosphorylation of PKR at Y162 regulates covalent PKR-SUMO2 interaction.

### Y162D mutation in PKR abolishes its activation by SUMO

**Figure 2:**
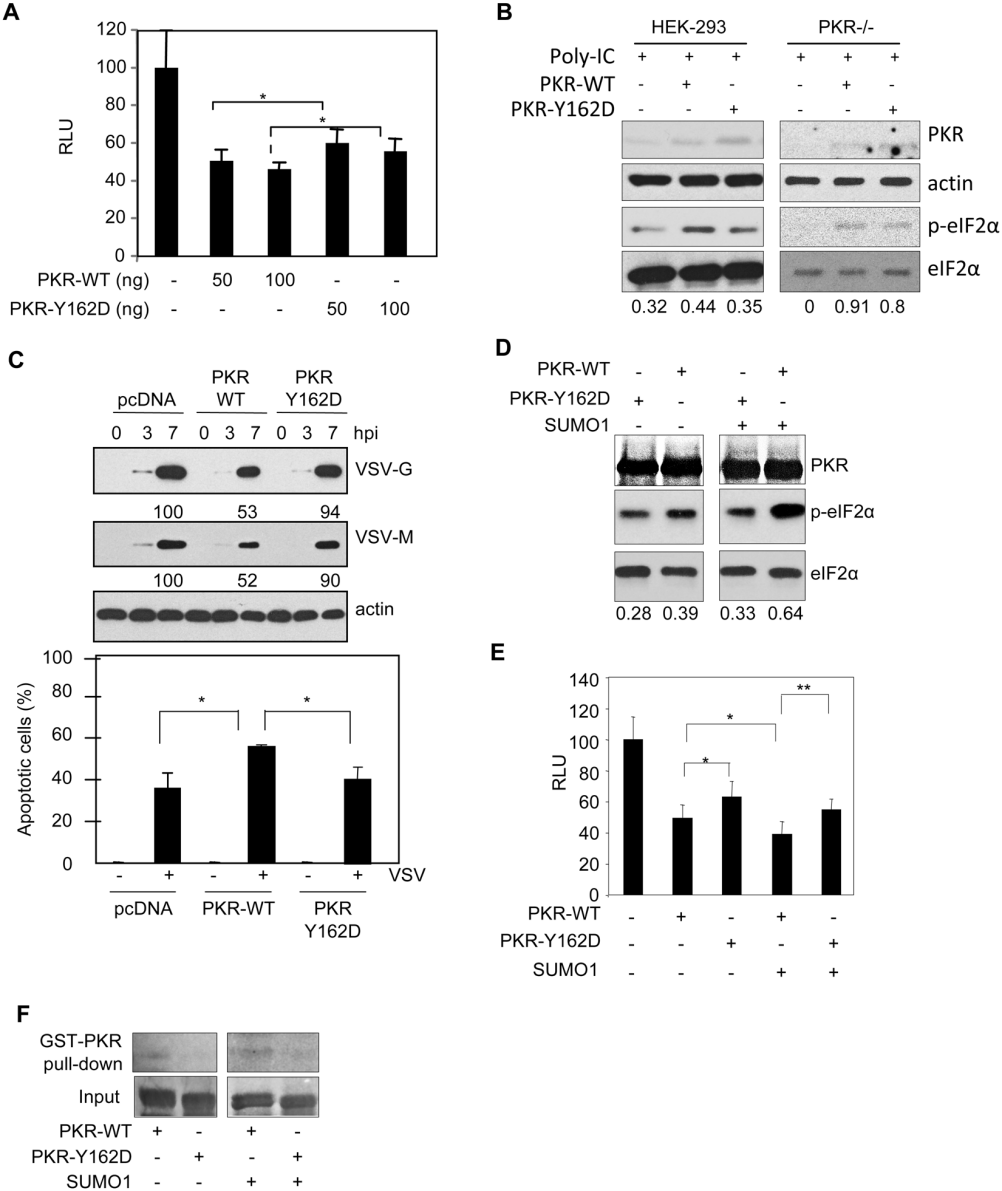
Y162D mutation in PKR abolishes its response to SUMO. (**A**) PKR−/− cells were co-transfected with the reporter plasmid PGL3-control together with the indicated plasmids, and then treated with poly(I:C) and 7 h after treatment were assayed for luciferase activity. The relative luciferase activity obtained after normalization to total protein amount is represented on the y-axis. Each experiment was done in triplicate and repeated three times. Bars, SE. *p < 0.05, Student’s t test. (**B**) HEK-293 cells (left panel) or PKR−/− cells (right panel) were transfected with the indicated plasmids, and then treated with poly(I:C) and 7 h after treatment cells were analyzed by Western-blot with the indicated antibodies. The values below the Western-blot panels represent the ratio of p-eIF2α/total eIF2α. (**C**) PKR−/− cells stably transfected with pcDNA, PKR-WT or PKR-Y162D were infected with VSV at a multiplicity of infection of 10, and at different times after infection cells were recovered and analyzed by Western-blot using anti-VSV-M and anti-VSV-G antibodies (upper panel). Quantification of VSV protein synthesis at 7 hpi normalized to the actin levels is shown below the VSV-G or VSV-M blots. An arbitrary level of 100 was assigned to cells transfected with pcDNA and the ratios for other samples were expressed as relative values. PKR−/− cells stably transfected with pcDNA, PKR-WT or PKR-Y162D were infected as described above and 24 h after infection were subjected to caspase staining according to the manufacturer specifications. Cells were then subjected to flow cytometry analysis by using FACScan. Each experiment was done in triplicate and repeated three times. Results are mean+/−SE from triplicates, *p < 0.05, Student’s t test. (**D**) *In vitro* kinase assay with *in vitro*-translated PKR-WT protein or the PKR-Y162D mutant previously subjected to *in vitro* SUMOylation assay in the presence or absence of SUMO1. Phosphorylation of eIF2α was detected using anti-phospho-eIF2α antibody. Samples from cropped blots are from the same experiment. The values below the Western-blot panels represent the ratio of p-eIF2α/total eIF2α. (**E**) PKR−/− cells were co-transfected with the reporter plasmid PGL3-control together with the indicated plasmids, treated with poly(I:C) and 7 h after treatment cells were assayed for luciferase activity. The relative luciferase activity obtained after normalization to total protein amount is represented on the y-axis. Each experiment was done in triplicate and repeated three times. Bars, SE. *p < 0.05; **p < 0.005, Student’s t test. (**F**) *In vitro*-translated [^35^S]methionine-labeled PKR-WT or PKR-Y162D proteins previously subjected to *in vitro* SUMOylation assay in the presence or absence of SUMO1 were tested for interaction with GST-PKR protein in the presence of poly(I:C).

Phosphorylation of PKR at tyrosine residues promotes the activity of the protein^3^, whereas inhibition of PKR SUMOylation reduces its capability to inhibit protein synthesis upon dsRNA treatment and to control VSV replication^8^. We thus decided to evaluate the activity of the Y162D mutant in the presence or absence of SUMO. PKR−/− cells were first co-transfected with PGL3-control together with 50 or 100 ng of PKR-WT or PKR-Y162D plasmids and 36 h after transfection cells were treated with dsRNA and luciferase expression was measured. The decrease in the luciferase reporter synthesis in the PKR-Y162D transfected cells was significantly lower than the inhibition detected in the PKR-WT transfected cells (Fig. 2A), indicative of a reduced efficiency of the mutant to control protein synthesis. We also carried out a Western-blot analysis of HEK-293 or PKR−/− cells transfected with PKR-WT or PKR-Y162D and treated with dsRNA, with antibody against phosphorylated eIF2α. As shown in Fig. 2B, the levels of phosphorylated eIF2α protein in cells expressing PKR-Y162D were lower than in PKR-WT cells. To confirm the reduced activity of PKR-Y162D mutant, we infected PKR−/− cells stably transfected with pcDNA, PKR-WT, or PKR-Y162D with VSV and analyzed the VSV protein synthesis and the apoptosis induced by the virus. We observed higher levels of VSV protein synthesis and lower levels of apoptosis in cells transfected with the PKR mutant than in the PKR-WT transfected cells (Fig. 2C). These results indicated that the tyrosine residue 162 in PKR is required for an efficient PKR activity. To evaluate whether this decreased activity of the Y162D mutant is due to its altered capability to interact with SUMO, we evaluated the effect of SUMO overexpression on the activity of the Y162D protein. First, we carried out an *in vitro* kinase assay using *in vitro*-translated PKR-WT or the PKR-Y162D mutant, and recombinant eIF2α as a substrate, in the presence or absence of SUMO1. As shown in Fig. 2D, PKR-WT induced the phosphorylation of eIF2α, and the incubation with SUMO1 increased the levels of phosphorylated eIF2α, as expected^8^. The levels of phosphorylated eIF2α detected using PKR-Y162D were slightly lower than the ones observed when we employed the PKR-WT protein and this difference was clearly increased in the presence of SUMO1 (Fig. 2D). These results suggested that phosphorylation of PKR at Y162 abolished the activation of PKR by SUMO. To further prove this hypothesis, PKR-deficient cells were co-transfected with PGL3-control and PKR-WT or PKR-Y162D, in the presence or absence of SUMO1, and 36 h after transfection cells were treated with dsRNA and luciferase expression was measured. As shown in Fig. 2E, the shut off of protein synthesis detected after transfection of PKR-Y162D was significantly lower than the one observed after PKR-WT transfection. Increasing the SUMOylation machinery components significantly enhanced the control of protein synthesis induced by PKR-WT but it did not significantly affect the control of protein synthesis mediated by PKR-Y162D (Fig. 2E).

Finally, in order to determine whether phosphorylation of PKR at Y162 also modulates PKR dimerization we decided to carry out a GST-PKR pulldown assay in the presence of dsRNA with *in vitro-*translated [^35^S]methionine-labeled PKR-WT or PKR-Y162D proteins previously subjected to an *in vitro* SUMOylation assay in the presence or absence of SUMO1. As shown in Fig. 2F, GST-PKR exhibited a stronger interaction with PKR-WT than with PKR-Y162D both, in the absence and in the presence of SUMO. All together these results indicate that phosphorylation of PKR at tyrosine residue Y162 represents a mechanism for determining PKR-SUMO interaction. Moreover, our results indicate that the non-covalent interaction with SUMO of PKR has an impact on PKR SUMOylation and, consequently, on PKR activity.

## Methods

### Cell lines and virus

3T3-like cells derived from homozygous PKR−/− mice were kindly provided by Dr. C. Weissmann. PKR−/− and HEK-293 cells were grown in DMEM supplemented with 10% FBS (Life Technologies), 5 mM L-glutamine (Life Technologies), and penicillin-streptomycin (Life Technologies). For infections, cells were infected with VSV of Indiana strain at a multiplicity of infection (MOI) of 10 pfu/cell.

### Plasmids, transfections, and reagents

Plasmid pcDNA3-PKR/HA^21^ coding for human PKR was a generous gift of Dr. B. Y. Ahn. Plasmids pcDNA-His6-SUMO1, pcDNA-His6-SUMO2, pcDNA-Ubc9, and pcDNA3-PKR/HA-SUMOmut were described previously^8,22,23^. Mutations were introduced using the QuikChange PCR-based site-directed mutagenesis kit (Stratagene, La Jolla, CA) according to the manufacturer’s instructions, using pcDNA3-PKR/HA plasmid as template and the oligonucleotides listed in Table 1. The cells were transfected using Xtreme (Roche Diagnostics) following manufacturer instructions. Recombinant eIF2α was purchased from ProSpec.

**Table 1:** Oligonucleotides used in site-directed mutagenesis.

Oligonucleotide	Sequence
Y101D-Forward	5′- GGATTATCCATGGGGAATGACATAGGCCTTATCAATAG -3′
Y101D-Reverse	5′- CTATTGATAAGGCCTATGTCATTCCCCATGGATAATCC -3′
Y101A-Forward	5′- GGATTATCCATGGGGAATGCCATAGGCCTTATCAATAG -3′
Y101A-Reverse	5′- CTATTGATAAGGCCTATGGCATTCCCCATGGATAATCC -3′
Y162D-Forward	5′- GGCCGCTAAACTTGCAGATCTTCAGATATTATCAG -3′
Y162D-Reverse	5′- CTGATAATATCTGAAGATCTGCAAGTTTAGCGGCC -3′
Y162A-Forward	5′- GGCCGCTAAACTTGCAGCTCTTCAGATATTATCAG -3′
Y162A-Reverse	5′- CTGATAATATCTGAAGAGCTGCAAGTTTAGCGGCC -3′
SIM102-Forward	5′-GGGAATTACATAGGCCTTGCCAATAGAATTGCCCAG-3′
SIM-102-Reverse	5′-CTGGGCAATTCTATTGGCAAGGCCTATGTAATTCCC-3′
SIM-163-Forward	5′-GCATATCTTCAGATAGCATCAGAAGAAACCTCAG-3′
SIM-163-Reverse	5'-CTGAGGTTTCTTCTGATGCTATCTGAAGATATGC-3′

### Generation of stable cell lines

PKR−/− cells were co-transfected with a plasmid DNA containing a puromycin resistance gene and the indicated PKR plasmids (1:10 ratio), and were selected with puromycin (3 µg/ml) for 3 days.

### *In vitro* SUMO conjugation assay

*In vitro* SUMO conjugation assays were performed on [^35^S]methionine-labeled *in vitro*-translated proteins as described previously^24^ using recombinant E1 SUMO-activating enzyme (SAE1/2) (Biomol, Enzo Life Sciences), E2 SUMO-conjugating enzyme (Ubc9), and SUMO1 or SUMO2. Proteins were separated by SDS-PAGE followed by autoradiography. The *in vitro* transcription/translation of proteins was performed by using 1 µg of plasmid DNA and a rabbit reticulocyte-coupled transcription/translation system following the manufacturer instructions (Promega).

### PKR protein kinase assay

Phosphorylation of eIF2α catalyzed by PKR wild-type or PKR-Y162D was carried out in the presence of dsRNA, and in the presence or absence of SUMO1, as indicated. The reaction was stopped by the addition of SDS-PAGE loading buffer, and proteins were separated by SDS-PAGE and transferred to nitrocellulose membrane. Phosphorylation of eIF2α was evaluated using the antibody anti-phospho-eIF2α.

### Purification of His-tagged conjugates and immunoprecipitation assays

The purification of His-tagged conjugates, using Ni^2+^-nitrilotriacetic acid-agarose beads allowing the purification of proteins that are covalently conjugated to His6-SUMO, was performed as described previously^25^. For immunoprecipitations, cells were lysed in RIPA buffer containing proteinase inhibitor mixture (Sigma) at 4 °C, cleared at 16,000 x g for 5 min and immunoprecipitated overnight at 4 °C after addition of 2 µl of the specified antibody and 50 µl of 50% protein A-Sepharose CL-4B beads (Amersham Biosciences). Beads were then washed 4 times with RIPA buffer and resuspended in 30 µl of SDS-PAGE loading buffer. Proteins were separated by SDS-PAGE and transferred to nitrocellulose membrane.

### Antibodies and Western blot analysis

Cells were washed in PBS, scraped in SDS-PAGE loading buffer and boiled for 5 min. Proteins of total extracts were separated by SDS-PAGE and transferred to nitrocellulose membrane. Western blotting was carried out using standard methods. All antibodies were used at a dilution of 1:1000 of the stock in blocking buffer (5% skim milk prepared in TBS-Tween). The membranes were incubated with the indicated antibodies, washed with TBS-Tween, and signals were detected by using chemiluminescence. Antibodies to PKR and eIF2α were purchased from Santa Cruz Biotechnology. Antibodies to phospho-eIF2α (Ser-51) and anti-SUMO2 were purchased from Life Technologies. Anti-VSV-M antibody was from KeraFAST. Anti-HA monoclonal antibody was purchased from Covance. Anti-actin antibody was from MP Biomedicals. Anti-phosphotyrosine antibody was from Cell Signaling. Anti-VSV-G antibody was a generous gift of Dr. I Ventoso.

### Luciferase reporter assay

Cells were co-transfected with the reporter PGL3-control and the indicated plasmids, and 36 h after transfection cells were incubated with poly(I:C) (5 µg/ml) for 7 h. Then, cell extracts were harvested and assayed for luciferase activity after normalizing for the transfection efficiency by measuring the total protein.

### GST pulldown

GST pulldown experiments with the recombinant GST-PKR protein were performed in the presence of poly(I:C) using [^35^S]methionine-labeled *in vitro*-translated PKR-WT or PKR-Y162D protein previously subjected to *in vitro* SUMOylation assay in the presence or absence of SUMO1, as described previously^8^. Pulldown experiments with GST-SUMO1 were performed as described previously^25^, using [^35^S]methionine-labeled *in vitro*-translated PKR WT or mutant proteins.

### Apoptosis quantification

Apoptosis was quantified by flow cytometry using the caspase-3, active form, mAb apoptosis kit from BD Pharmingen, according to the manufacturer instructions.

### Statistical analysis

For statistical analysis between control and different groups, the Student’s t test was applied. The significance level chosen for the statistical analysis was p < 0.05.

## Electronic supplementary material

Supplementary information

## References

[1] Garcia MA (2006). Impact of protein kinase PKR in cell biology: from antiviral to antiproliferative action. Microbiol Mol Biol Rev.

[2] Romano PR (1998). Autophosphorylation in the activation loop is required for full kinase activity *in vivo* of human and yeast eukaryotic initiation factor 2alpha kinases PKR and GCN2. Mol Cell Biol.

[3] Su Q (2006). Tyrosine phosphorylation acts as a molecular switch to full-scale activation of the eIF2alpha RNA-dependent protein kinase. Proc Natl Acad Sci USA.

[4] Taylor DR (1996). Autophosphorylation sites participate in the activation of the double-stranded-RNA-activated protein kinase PKR. Mol Cell Biol.

[5] Hovanessian AG, Galabru J (1987). The double-stranded RNA-dependent protein kinase is also activated by heparin. Eur J Biochem.

[6] Okumura F (2013). Activation of double-stranded RNA-activated protein kinase (PKR) by interferon-stimulated gene 15 (ISG15) modification down-regulates protein translation. J Biol Chem.

[7] Patel RC, Sen GC (1998). PACT, a protein activator of the interferon-induced protein kinase, PKR. Embo J.

[8] de la Cruz-Herrera CF (2014). Activation of the double-stranded RNA-dependent protein kinase PKR by small ubiquitin-like modifier (SUMO). J Biol Chem.

[9] Lin DY (2006). Role of SUMO-interacting motif in Daxx SUMO modification, subnuclear localization, and repression of sumoylated transcription factors. Mol Cell.

[10] Marcos-Villar L (2011). Covalent modification by SUMO is required for efficient disruption of PML oncogenic domains by Kaposi’s sarcoma-associated herpesvirus latent protein LANA2. J Gen Virol.

[11] Song J, Durrin LK, Wilkinson TA, Krontiris TG, Chen Y (2004). Identification of a SUMO-binding motif that recognizes SUMO-modified proteins. Proc Natl Acad Sci USA.

[12] Takahashi H, Hatakeyama S, Saitoh H, Nakayama KI (2005). Noncovalent SUMO-1 binding activity of thymine DNA glycosylase (TDG) is required for its SUMO-1 modification and colocalization with the promyelocytic leukemia protein. J Biol Chem.

[13] Gareau JR, Lima CD (2010). The SUMO pathway: emerging mechanisms that shape specificity, conjugation and recognition. Nat Rev Mol Cell Biol.

[14] Kerscher O (2007). SUMO junction-what’s your function? New insights through SUMO-interacting motifs. EMBO Rep.

[15] Meulmeester E, Melchior F (2008). Cell biology: SUMO. Nature.

[16] Minty A, Dumont X, Kaghad M, Caput D (2000). Covalent modification of p73alpha by SUMO-1. Two-hybrid screening with p73 identifies novel SUMO-1-interacting proteins and a SUMO-1 interaction motif. J Biol Chem.

[17] Zhu H, Zhou JN (2008). SUMO1 enhances 17-beta estradiol’s effect on CRH promoter activation through estrogen receptors. Neuro Endocrinol Lett.

[18] Cappadocia L (2015). Structural and functional characterization of the phosphorylation-dependent interaction between PML and SUMO1. Structure.

[19] Hecker CM, Rabiller M, Haglund K, Bayer P, Dikic I (2006). Specification of SUMO1- and SUMO2-interacting motifs. J Biol Chem.

[20] Song J, Zhang Z, Hu W, Chen Y (2005). Small ubiquitin-like modifier (SUMO) recognition of a SUMO binding motif: a reversal of the bound orientation. J Biol Chem.

[21] Kang JI (2009). PKR protein kinase is activated by hepatitis C virus and inhibits viral replication through translational control. Virus Res.

[22] Desterro JM, Rodriguez MS, Hay RT (1998). SUMO-1 modification of IkappaBalpha inhibits NF-kappaB activation. Mol Cell.

[23] Vertegaal AC (2006). Distinct and overlapping sets of SUMO-1 and SUMO-2 target proteins revealed by quantitative proteomics. Mol Cell Proteomics.

[24] Campagna M (2011). SIRT1 stabilizes PML promoting its sumoylation. Cell Death Differ.

[25] Marcos-Villar L (2009). Kaposi’s sarcoma-associated herpesvirus protein LANA2 disrupts PML oncogenic domains and inhibits PML-mediated transcriptional repression of the survivin gene. J Virol.

