# Supplementary material for: Phosphorylable tyrosine residue 162 in the double-stranded RNA-dependent kinase PKR modulates its interaction with SUMO
Source: Sci Rep. 2017 Oct 25;7:14055. doi: 10.1038/s41598-017-12777-7 (PMC5656663; doi:10.1038/s41598-017-12777-7)
Supplement: Supplementary file 1 — Supplementary information [file 41598_2017_12777_MOESM1_ESM.pdf]

Supplementary information

Phosphorylable tyrosine residue 162 in the double-stranded RNA-dependent kinase PKR modulates its interaction with SUMO

Carlos F de la Cruz-Herrera, Maite Baz-Martínez, Ahmed El Motiam, Santiago Vidal, Manuel Collado, Anxo Vidal, Manuel S Rodríguez, Mariano Esteban, and Carmen Rivas

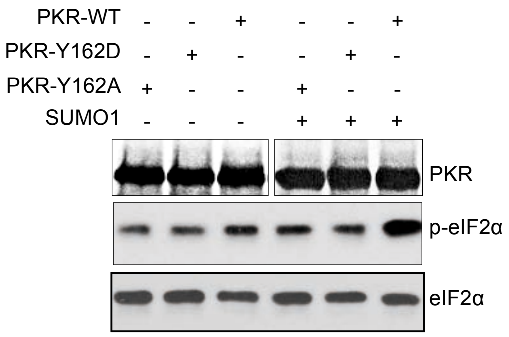

Uncropped anti- p-eiF2a and eIF2a blots shown in Figure 2D

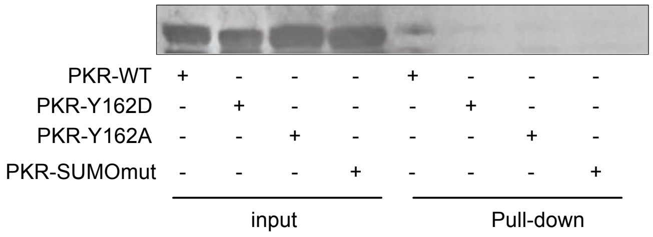

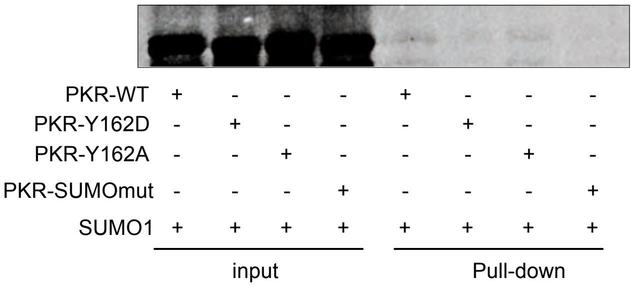

Uncropped blots showing input and pull-down samples from Figure 2F

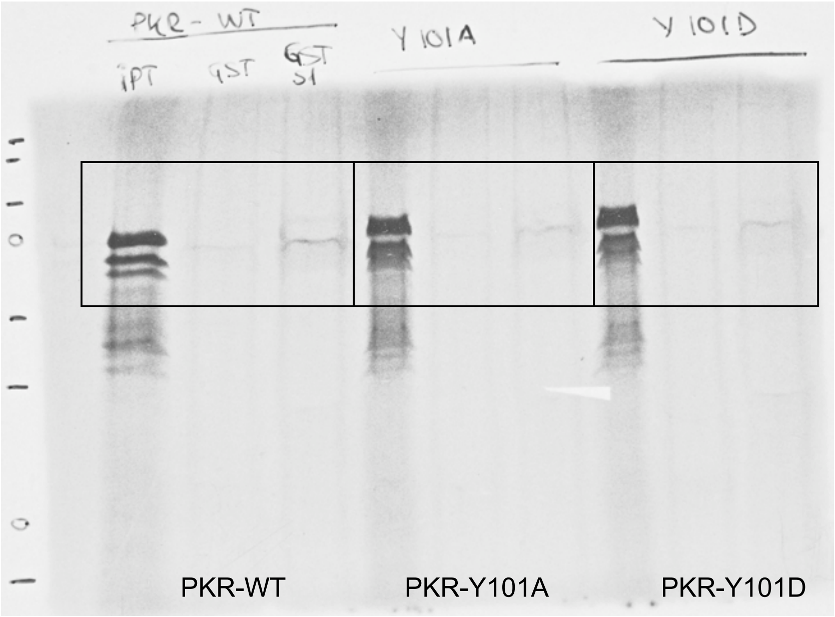

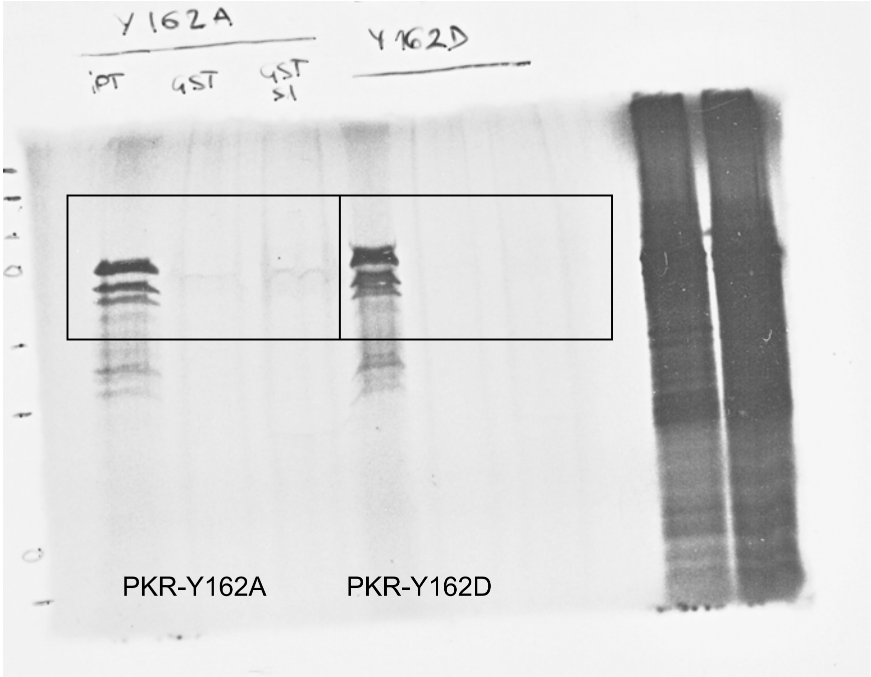

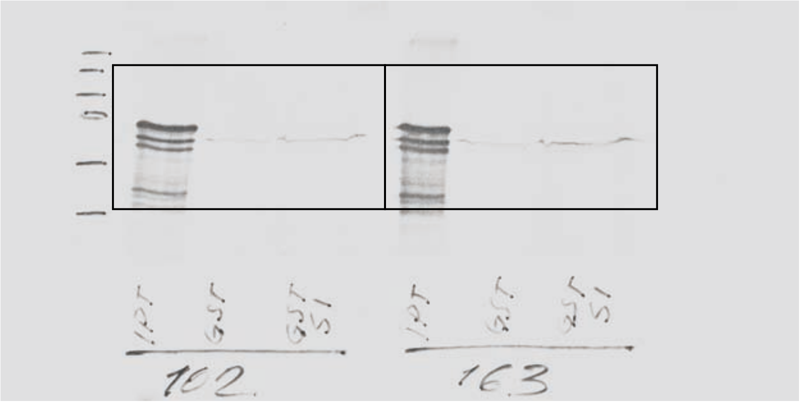

PKR-Y102 PKR-Y163

Original Western-blot gels associated with Figure 1B

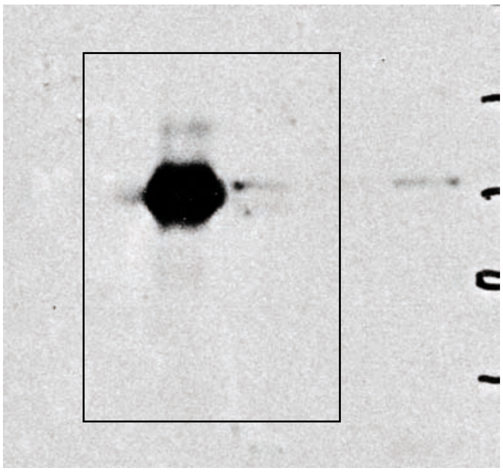

Original Western-blot gel associated with Figure 1F
